# Insights Into Factors Affecting Nurses’ Knowledge of and Attitudes Toward AI and Implications for Successful AI Integration in Critical Care: Cross-Sectional Study

**DOI:** 10.2196/85649

**Published:** 2026-01-16

**Authors:** Habib Alrashedi, Saad M Alderaan, Nader Alnomasy, Hamdi Lamine, Khalil A Saleh, Sameer A Alkubati

**Affiliations:** 1 Medical-Surgical Nursing Department, College of Nursing, University of Ha'il, Baqaa street, Hail, 55439, Saudi Arabia, 966 507306911; 2 Hail Health Cluster, King Khaled Hospital, Hail, Saudi Arabia; 3 Community Health Nursing Department, College of Nursing, University of Ha'il, Hail, Saudi Arabia

**Keywords:** artificial intelligence, AI, knowledge, attitudes, factors, critical care nurses, Saudi Arabia

## Abstract

**Background:**

Assessing the current landscape of nurses’ knowledge and attitudes is a critical first step in facilitating a smooth and effective transition toward artificial intelligence (AI)–enhanced critical care.

**Objective:**

This study aimed to assess the levels of and factors affecting the knowledge of and general attitudes toward AI in critical care among nurses.

**Methods:**

A cross-sectional correlational design was used with 203 critical care nurses in Hail, Saudi Arabia, using the Nurses’ AI Knowledge Questionnaire and the 20-item General Attitudes Toward Artificial Intelligence Scale from May 2025 to July 2025. Data were analyzed using 2-tailed *t* tests, ANOVA, Pearson correlation, and multivariable linear regression. Statistical significance was set at *P*<.05.

**Results:**

Critical care nurses demonstrated moderate knowledge of (mean score 4.93, SD 1.78) and positive attitudes toward AI (mean score 64.39, SD 8.26). A moderate positive correlation was found between knowledge of and attitudes toward AI (*r*=0.45; *P*<.001). In multivariable analyses, older age was associated with lower knowledge (≥40 years: β=−1.29, 95% CI −2.12 to −0.45; *P*=.003) and less positive attitudes (β=−8.97, 95% CI −12.49 to −5.44; *P*<.001). Female nurses reported lower knowledge (β=−0.69, 95% CI −1.20 to −0.19; *P*=.007) and less positive attitudes (β=−2.65, 95% CI −4.78 to −0.52; *P*=.02) than male nurses. Greater experience (>5 years) was positively associated with knowledge (β=1.20, 95% CI 0.65‐1.75; *P*<.001) and attitudes (β=8.08, 95% CI 5.76‐10.41; *P*<.001).

**Conclusions:**

Critical care nurses in Hail demonstrated moderate knowledge of and positive attitudes toward AI, which varied based on their demographic and professional characteristics. These findings highlight the need to strengthen AI literacy and provide targeted support to groups with lower scores, which may enhance readiness for AI integration in critical care settings.

## Introduction

The global health care model is undergoing a great revolution directed by the rapid incorporation of artificial intelligence (AI) [1,2]. AI is defined as a system designed to perceive the environment and take action to achieve specific goals [3]. AI encompasses machine learning, natural language processing, and robotics [2,4]. In medicine, AI has vast and promising potential for enhancing diagnostic precision; personalizing treatment plans; optimizing operational efficiency; and, ultimately, improving patient outcomes [5,6]. This technological shift aligns with global initiatives such as Vision 2030, which actively encourages innovation and digital transformation within its health sector to build a robust, data-driven health care system [7,8].

Critical care units, including intensive care units and emergency departments, represent environments with exceptionally high pressure [9]. Clinicians in these settings are required to process large amounts of complex, real-time patient data to make swift decisions. AI applications can be leveraged for the early detection of patient deterioration, prediction of sepsis, forecasting intensive care unit length of stay, and managing ventilatory support. These capabilities support clinical decision-making and potentially reduce human burden [10]. By automating routine tasks and administrative burdens, AI can free critical care nurses (CCNs) and physicians to focus on more complex clinical reasoning and direct patient care. This shift enhances both the efficiency and humanistic aspects of treatment [11].

As frontline clinicians, nurses are pivotal to the successful adoption of new technologies in clinical practice [12]. Their role involves continuous patient monitoring, assessment, and execution of complex care plans, making them key end users of AI-driven tools. Therefore, the effective integration of AI into critical care is linked to nurses’ acceptance, which is shaped by their knowledge, attitudes, and willingness to incorporate these technologies into their workflow [13,14]. However, the introduction of AI in the nursing domain has sparked debate. While some view AI as a tool to augment nursing practice and mitigate workload, others perceive it as a threat to the essential human-to-human interactions that form the bedrock of compassionate care. This difference in perspective raises concerns about dehumanization and ethical implications [15-17].

A significant barrier to AI’s integration is the current underrepresentation of nurses in the development, implementation, and evaluation of AI systems for health care [18,19]. This gap can lead to a misalignment among technological solutions, actual clinical needs, and workflow. Furthermore, studies have indicated that nurses’ perceptions of AI are mixed and vary widely based on their understanding of its capabilities, reliability, and potential to replace human judgment [20]. Therefore, assessing the current landscape of nurses’ knowledge and attitudes is a critical first step in facilitating a smooth and effective transition toward AI-enhanced critical care.

Previous research has begun to explore health care professionals’ perspectives on AI, but studies focused on CCNs within the Middle Eastern context, particularly in Saudi Arabia, remain limited. Understanding the demographic, educational, and experiential factors that influence these perceptions is crucial for developing targeted educational and training programs. As critical care environments become increasingly technologically advanced, ensuring that the nursing workforce is not only proficient but also confident and ethically grounded in using AI is paramount.

This study aimed to bridge this knowledge gap by assessing CCNs’ level of knowledge of and general attitudes toward AI in Hail, Saudi Arabia. The findings provide valuable insights for hospital administrators, educators, and policymakers in designing strategies that foster AI literacy and address concerns. The ultimate goal is to harness the full potential of AI to support rather than replace the critical role of nurses in delivering high-acuity patient care.

## Methods

### Design, Setting, Population, and Sample

A cross-sectional correlational design was used in this study. The target population of this study comprised CCNs employed in public hospitals located in the Hail region. After obtaining institutional review board approval, meetings were conducted with the heads of critical care units across participating hospitals. In addition, formal communication was established with continuing nursing education offices within these institutions. The survey was designed using Google Forms. The link to the questionnaire and informed consent form was disseminated to the CCNs. Participants were first given an electronic information sheet outlining the study’s goals, procedures, risks, benefits, confidentiality measures, and voluntary nature of participation. Data collection was conducted over 3 months, from May 2025 to July 2025. Eligibility criteria required participants to have at least 1 year of continuous experience working in critical care departments, ensuring that only nurses with sufficient exposure to clinical practice in critical situations were included. Nurses serving primarily in administrative roles, as well as those with less than 1 year of critical care department experience, were excluded to maintain a focus on direct patient care providers with adequate professional backgrounds. The required sample size was determined using OpenEpi (version 3.01). On the basis of an estimated total population of approximately 420 CCNs in the region, the minimum sample size necessary to achieve adequate statistical power was 201, with a 95% confidence level and 5% margin of error. To enhance representativeness and mitigate the potential impact of nonresponses, 220 self-administered questionnaires were distributed. Of these 220 questionnaires, 203 (92.3%) were returned. The electronic survey required responses to all the items before submission; therefore, there were no missing data.

### Instruments

The questionnaire consisted of 3 parts. In the first part, the characteristics of the nurses, including their age, sex, marital status, educational level, years of experience, type of shift work, unit type, and prior experience with AI in health care, were examined. We used a previously validated tool by Swed et al [21] (the Nurses’ AI Knowledge Questionnaire) in the second segment of the questionnaire to gauge nurses’ awareness of AI. It consists of 7 yes-or-no questions regarding common AI terms used in health care designed to gauge nurses’ familiarity with this key vocabulary. The scoring system is as follows: “yes” answers are scored as 1 point, and “no” answers are scored as 0 points. The total score ranges from 0 to 7, with a higher score indicating a higher level of knowledge regarding AI terminology [21]. As Swed et al [21] noted, the Cronbach α value of 0.795 demonstrated the tool’s internal consistency among the subscales. In this study, the reliability of this instrument was confirmed with a Cronbach α of 0.765.

The 20-item General Attitudes Toward Artificial Intelligence Scale (GAAIS) created by Schepman and Rodway [22] constituted the third section of the questionnaire. It gauged nurses’ opinions on the use of AI in medical environments. The items are divided into positive (12 items) and negative (8 items). Positive items are scored on a 5-point Likert-type scale, with 1 denoting “strongly disagree” and 5 denoting “strongly agree.” Negative items are reverse scored, with 1 denoting “strongly agree” and 5 denoting “strongly disagree.” Thus, the scores range from 20 to 100, with higher scores on each subscale reflecting more positive attitudes [22]. According to Schepman and Rodway [22], the GAAIS has demonstrated a high degree of internal consistency, with Cronbach α values for the 12 positive items and 8 negative items being 0.88 and 0.82, respectively. In this study, reliability was confirmed with a Cronbach α of 0.969 for positive items and 0.952 for negative items.

### Ethical Considerations

Institutional review board approval was obtained from the University of Hail (H-2025-718) on March 10, 2025, and from the Ministry of Health (2025-37) on March 18, 2025. In compliance with institutional review board approval, informed consent was obtained electronically: participants were required to study the information page before completing and submitting the survey, which constituted informed consent. The participants’ anonymity and confidentiality were maintained throughout the study. As the survey platform required all items to be completed before submission, there were no partial responses or missing data. No compensation or incentives were provided for participation in the study.

### Data Analysis

SPSS Statistics (version 27; IBM Corp) was used to analyze the data. The Shapiro-Wilk test was used to test for normality of the data (*P*>.05). Independent-sample *t* tests and one-way ANOVA were used to investigate the relationship between the dependent and independent variables. Using multivariable linear regression analysis, significant factors affecting CCNs’ knowledge and attitudes were identified. Correlations between variables were assessed using the Pearson correlation coefficient. The *P* value was set at less than .05.

## Results

Table 1 shows that most participants were aged 20 to 29 years (121/203, 59.6%), male (129/203, 63.5%), and single (140/203, 69%). Most nurses held a bachelor’s degree (141/203, 69.5%), worked rotating shifts (131/203, 64.5%), and had 5 years or less of nursing experience (117/203, 57.6%). Younger nurses showed significantly higher knowledge of (*P*=.01) and more positive attitudes toward AI (*P*=.002) than older nurses. Male nurses reported higher knowledge and more positive attitudes than female nurses (*P*<.001 in both cases). Single nurses scored higher on knowledge (*P*=.03) and attitudes (*P*=.046) than married nurses. Nurses with a master’s degree had higher knowledge (*P*=.02) and more positive attitudes (*P*<.001) than those with a bachelor’s degree. Additionally, nurses with more than 5 years of experience exhibited higher knowledge (*P*=.02) and more positive attitudes (*P*<.001) than less experienced nurses.

**Table 1.** Relationship between critical care nurses’ (CCNs) sociodemographic characteristics and knowledge of and attitudes toward artificial intelligence (N=203).

Variable and categories	CCNs, n (%)	Knowledge			Attitudes		
		Score (0-7), mean (SD)	*t* test (*df*) or *F* test (*df*)	*P* value	Score (20-100), mean (SD)	*t* test (*df*) or *F* test (*df*)	*P* value
Age (years)			4.61 (2)^a^	.01		6.55 (2)^a^	.002
20-29	121 (59.6)	5.20 (1.68)			65.60 (8.16)		
30-39	60 (29.6)	4.68 (1.94)			63.96 (8.44)		
≥40	22 (10.8)	4.09 (1.60)			58.90 (5.99)		
Sex			3.78 (201)^b^	<.001		3.54 (201)^b^	<.001
Male	129 (63.5)	5.27 (1.65)			65.90 (8.32)		
Female	74 (36.5)	4.32 (1.85)			61.75 (7.53)		
Marital status			2.20 (201)^b^	.03		1.88 (201)^b^	.046
Single	140 (69.0)	5.11 (1.71)			65.12 (8.62)		
Married	63 (31.0)	4.52 (1.88)			62.77 (7.22)		
Educational level			−2.35 (201)^b^	.02		−4.02 (201)^b^	<.001
Bachelor’s	141 (69.5)	4.73 (1.80)			62.90 (7.17)		
Master’s	62 (30.5)	5.37 (1.68)			67.79 (9.56)		
Shift type			−0.41 (201)^b^	.68		−1.09 (201)^b^	.28
Day	72 (35.5)	4.86 (1.78)			63.54 (7.79)		
Rotating	131 (64.5)	4.96 (1.79)			64.86 (8.51)		
Experience (years)			−2.41 (201)^b^	.02		−4.38 (201)^b^	<.001
≤5	117 (57.6)	4.67 (1.78)			62.30 (6.91)		
>5	86 (42.4)	5.27 (1.73)			67.23 (9.12)		

a *F* test.

b 2-tailed *t *test.

The mean score for CCNs’ knowledge of AI was 4.93 (SD 1.78; range 2‐7), indicating a moderate level of knowledge. The mean score for attitudes was 64.39 (SD 8.26; range 47‐95), reflecting a generally positive attitude toward AI (Table 2).

Table 3 shows that there was a moderate positive correlation between CCNs’ knowledge of and attitudes toward AI (*r*=0.45; *P*<.001). This finding indicates that higher knowledge levels are associated with more positive attitudes.

**Table 2.** Means of critical care nurses’ knowledge of and attitudes toward artificial intelligence (N=203).

Variable	Score, mean (SD; range)
Knowledge (0-7)	4.93 (1.78; 2-7)
Attitudes (20-100)	64.39 (8.26; 47-95)

**Table 3.** Correlation between study variables (N=203).

	Knowledge	Attitudes
Knowledge	1	0.45
Attitudes	0.45^a^	1

a Correlation is significant at the .01 level (2 tailed).

**Figure 1.**
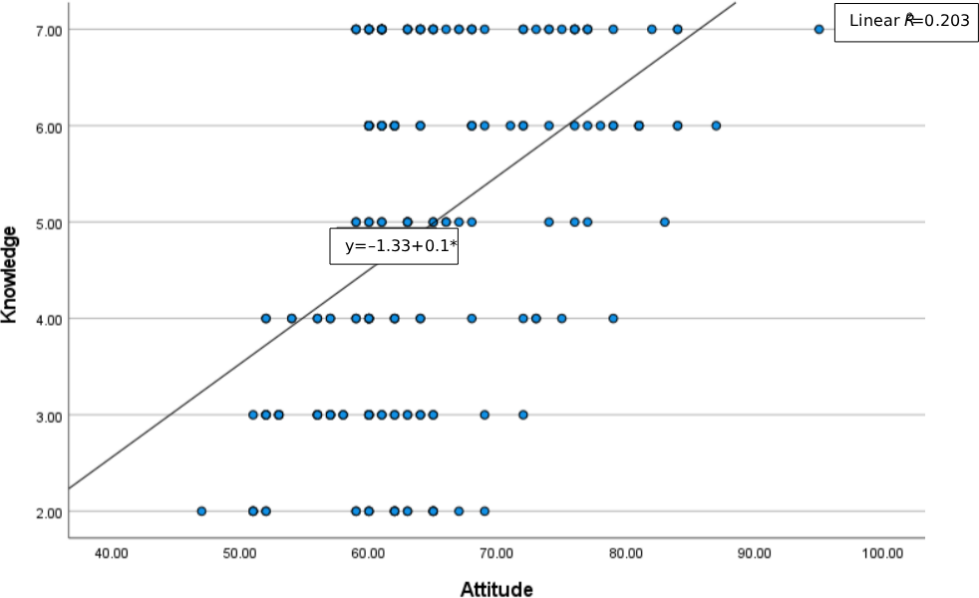
Scatterplot of the correlation between critical care nurses’ knowledge of and attitudes toward artificial intelligence.

Figure 1 illustrates the moderate positive correlation between CCNs’ knowledge of and attitudes toward AI. The scatterplot shows that higher attitude scores were associated with higher knowledge levels. The regression line indicates a significant positive linear relationship (*R*^2^=0.20, *P*<.001), suggesting that attitudes were associated with approximately 20% of the variance in knowledge.

The regression models identified several sociodemographic predictors of CCNs’ knowledge of and attitudes toward AI. Age and sex were found to be significant predictors of knowledge. Nurses aged 30 to 39 years (β=–0.80; *P*=.02) and those aged ≥40 years (β=–1.29; *P*=.003) had lower knowledge scores than those aged 20 to 29 years. Similarly, female nurses reported significantly lower knowledge scores than their male counterparts (β=−0.69; *P*=.007). In contrast, nurses with more than 5 years of experience had significantly higher knowledge levels (β=1.20; *P*<.001). The model’s *R*^2^ was 0.19 (adjusted *R*^2^=0.17; *P*<.001), indicating that the included predictors were associated with approximately 19.4% of the variability in knowledge scores.

Age, sex, educational level, and experience were significant predictors of attitudes. Nurses aged 30 to 39 years (β=–4.81; *P*=.001) and those aged ≥40 years (β=–8.97; *P*<.001) reported less positive attitudes than those aged 20 to 29 years. Female nurses had significantly less positive attitudes than male nurses (β=–2.65; *P*=.02). Conversely, nurses with a master’s degree (β=3.38; *P*=.002) and those with more than 5 years of experience (β=8.08; *P*<.001) demonstrated more positive attitudes. The model’s *R*^2^ was 0.33 (adjusted *R*^2^=0.31; *P*<.001), indicating that the included predictors were associated with approximately 33.2% of the variability in attitude scores (Table 4).

**Table 4.** Multiple linear regression for factors affecting critical care nurses’ knowledge of and attitudes toward artificial intelligence.

Factor	Knowledge^a^		Attitudes^b^	
	β (95% CI)	*P* value	β (95% CI)	*P* value
Age (years)				
20-29	Reference	Reference	Reference	Reference
30-39	−0.80 (−1.47 to –0.14)	.02	−4.81 (−7.59 to –2.02)	.001
≥40	−1.29 (−2.12 to –0.45)	.003	−8.97 (−12.49 to –5.44)	<.001
Gender				
Male	Reference	Reference	Reference	Reference
Female	−0.69 (−1.20 to –0.19)	.007	−2.65 (−4.78 to –0.52)	.02
Marital status				
Single	Reference	Reference	Reference	Reference
Married	−0.44 (−0.98 to 0.10)	.11	−1.73 (−4.01 to 0.55)	.14
Educational level				
Bachelor’s degree	Reference	Reference	Reference	Reference
Master’s degree	0.40 (−0.10 to 0.90)	.12	3.38 (1.270 to 5.492)	.002
Experience (years)				
≤5	Reference	Reference	Reference	Reference
>5	1.20 (0.65 to 1.75)	<.001	8.08 (5.76 to 10.41)	<.001

a *R*2=0.19; adjusted *R*2=0.17; *P*<.001.

b *R*2=0.33; adjusted *R*2=0.31; *P*<.001.

## Discussion

### CCNs’ Knowledge of and Attitudes Toward AI

This study provides a timely investigation of CCNs’ knowledge of and attitudes toward AI in the Hail region of Saudi Arabia, a context undergoing rapid digital transformation as part of Vision 2030. The findings revealed a moderate level of AI knowledge (mean score 4.93, SD 1.78) and a generally positive attitude (mean score 64.39, SD 8.26) among CCNs. Crucially, a significant positive correlation was established, indicating that higher levels of AI knowledge were associated with more favorable attitudes. This aligns with the technology acceptance model (TAM), which posits that perceived usefulness and ease of use are key determinants of technology adoption and that these perceptions are inherently linked to an individual’s understanding of the technology [23-25]. Our results suggest that educational interventions aimed at improving AI literacy could be a powerful lever for enhancing acceptance among the nursing workforce.

### Predictors of CCNs’ Knowledge of and Attitudes Toward AI

Sociodemographic analyses yielded insightful results. Younger nurses (aged 20-29 years) exhibited significantly higher knowledge and more positive attitudes than their older counterparts. This generational divide is consistent with the broader literature on technology adoption, where younger individuals, often “digital natives,” tend to be more comfortable and familiar with emerging technologies [26,27]. This finding underscores the need for age-tailored training programs that support more experienced nurses in developing comparable levels of perceived ease of use and usefulness, thereby reducing TAM-related barriers among older staff while leveraging their clinical expertise.

Gender emerged as a significant predictor, with male nurses reporting higher knowledge and more positive attitudes than female nurses. This disparity may reflect broader societal and educational trends in the science, technology, engineering, and mathematics fields, where gender gaps in confidence and participation persist [28]. In the nursing context, which is predominantly female in many countries but has a different demographic profile in regions such as Saudi Arabia, this finding underscores the need for equitable access to AI training and leadership opportunities. Ensuring that these opportunities are encouraging and available to all genders is essential to preventing a new form of digital gender divide within the profession. From a TAM perspective, such differences may translate into unequal perceptions of ease of use and self-efficacy with AI systems, highlighting the importance of designing AI training and leadership opportunities that actively foster confidence and perceived control among women to prevent a digital gender divide in nursing.

Educational attainment has a strong positive influence. Nurses with a master’s degree had significantly more positive attitudes and higher knowledge scores than those with a bachelor’s degree. This finding reinforces the pivotal role of advanced education in fostering a forward-looking, evidence-based, and innovative mindset. This suggests that integrating AI concepts and applications into postgraduate nursing curricula is essential for preparing future nurse leaders [6,29]. Furthermore, contrary to what might be assumed, nurses with more than 5 years of experience reported higher knowledge and more positive attitudes. This compelling finding challenges the notion that experienced clinicians are resistant to change. Instead, it implies that experienced nurses, with their developed clinical expertise, may better appreciate AI’s potential to alleviate cognitive burdens, reduce errors, and enhance patient safety [11].

The regression models indicated that 19.4% of the variance in knowledge and 33.2% of the variance in attitudes were related to demographic factors. However, other variables not measured in this study also played a significant role. These could include organizational culture, quality of previous technology implementation experiences, perceived organizational support for training, and the level of trust in the institution’s data governance and ethical frameworks [30,31]. Future research should explore these organizational and psychological determinants to provide a more holistic understanding of the factors that influence AI integration in nursing.

### Correlation Between CCNs’ Knowledge of and Attitudes Toward AI

The moderate positive correlation between knowledge and attitude (*r*=0.45; *P*<.001) strongly implies that resistance or skepticism toward AI is not unchangeable but can be mitigated through education and exposure. Within the TAM framework, this implies that structured education and meaningful hands-on experience can reshape nurses’ beliefs about AI’s usefulness and ease of use, moving them from passive compliance to active, informed adoption of AI tools in clinical workflows. This finding aligns with the work by Dornan [32], who suggested that a basic understanding of AI is essential for its acceptance and use in clinical practice. When nurses understand how AI works, what it can offer, and what its limits are, the technology becomes less intimidating [32]. This awareness helps them move from being passive recipients of change to being active and informed participants. Such a shift encourages genuine engagement rather than simple compliance [32]. Therefore, the observed link highlights the need for targeted education programs designed to build nurses’ confidence and skills in working with AI, ensuring that technology, rather than distance, enhances nursing care. Thus, the primary barrier is not an inherent opposition to technology but a lack of structured and accessible education on what AI truly entails in nursing practice.

### Implications for Nursing Education and Practice

The findings underscore the need for specific measures to improve AI preparedness among nurses through education. Older, female, and less experienced nurses had less knowledge of and a negative attitude toward AI, suggesting possible gaps in confidence and exposure that must be addressed through systematic training activities. Incorporating fundamental AI principles and practical applications into undergraduate and postgraduate nursing courses is critical for ensuring that all future nurses are prepared to work with developing technology. In nursing practice, continuing professional development programs that include practical training, simulation-based learning, and case-based scenarios can help improve comprehension and minimize anxiety. Furthermore, nursing professionals with a more optimistic mindset can act as clinical representatives to assist in collaborative learning and ensure the smooth integration of AI in critical care settings. Improving AI literacy across the nursing profession will eventually lead to safer and more efficient clinical practice and successful incorporation of AI-driven strategies in patient care.

These findings are particularly important in the context of Saudi Arabia’s ambitious health sector reforms. For AI to be successfully leveraged to build a robust, data-driven health care system as envisioned in Vision 2030, the readiness of the nursing workforce is fundamental. Aligning educational strategies, professional development, and organizational policies with TAM principles by explicitly targeting perceived usefulness, ease of use, and supportive conditions can help translate the current foundational willingness among nurses into sustained, confident use of AI in everyday practice. Our study confirms that foundational willingness is present but must be actively cultivated through targeted, demographically sensitive, and continuous educational strategies.

### Strengths and Limitations

This study has several strengths. First, it addresses a significant gap in the literature by focusing specifically on CCNs in the underresearched Middle Eastern context, thus providing valuable insights for regional policymaking and educational planning. The use of validated instruments such as the Nurses’ AI Knowledge Questionnaire and the GAAIS enhances the reliability and validity of the findings. Furthermore, the high response rate (203/220, 92.3% returned questionnaires) and rigorous sample size calculation strengthened the statistical power and representativeness of the results for the target population in Hail.

Despite these strengths, this study has several limitations that must be acknowledged. The cross-sectional correlational design captures a snapshot in time and cannot establish causality between the variables. This study was conducted in a single region of Saudi Arabia (Hail), which may limit the generalizability of the findings to other regions or countries with different cultural and health care infrastructures. The reliance on self-reported data for knowledge and attitudes is susceptible to social desirability bias, in which participants may have provided answers that they believed were expected rather than their true beliefs. Finally, the knowledge assessment was based on a 7-item yes-or-no questionnaire, which, while reliable, may not capture the full depth and nuance of a nurse’s understanding of AI concepts and applications.

### Recommendations

On the basis of this study’s findings, the following recommendations are proposed.

#### Recommendations for Practice

Health care institutions should implement structured, ongoing AI education programs that build basic literacy for all CCNs and provide advanced modules for those in higher-responsibility roles. These programs should use practical, critical care examples to enhance perceived usefulness and ease of use. Targeted support is also needed for older and female nurses who showed lower knowledge and more negative attitudes through tailored workshops, mentorship, and simulation-based training to ensure equitable AI readiness. Additionally, nurses with master’s degrees and more than 5 years of experience should be leveraged as AI champions to mentor colleagues and support effective implementation across critical care teams.

#### Recommendations for Education

Nursing schools and universities should integrate core AI content—concepts, clinical applications, ethics, and limitations—into undergraduate curricula while expanding advanced, practice-oriented AI training in postgraduate programs, reflecting the strong association between higher education and more positive perceptions. Alignment between academic preparation and workplace training will create a continuous pipeline of nurses who are both knowledgeable about and favorably disposed toward AI in critical care.

#### Recommendations for Policy

Hospital leadership and AI developers should systematically involve CCNs from diverse age groups, genders, and educational backgrounds in the design, piloting, and evaluation of AI tools because the regression results suggest that attitudes and knowledge are shaped partly by contextual and experiential factors beyond demographics alone. Participatory implementation can improve the perceived relevance and usability of AI systems, thereby reinforcing the positive relationship between knowledge and attitudes and supporting Saudi Arabia’s Vision 2030 for a technology-driven, nurse-ready health care system.

### Conclusions

This study demonstrated that CCNs in Hail, Saudi Arabia, possess a moderate level of knowledge and a generally positive attitude toward AI, with a clear correlation between the 2. Key sociodemographic factors, including age, sex, educational level, and clinical experience, significantly influenced these perceptions. The findings underscore that the successful integration of AI into critical care is not a technological challenge but a human-centric one. Readiness of the nursing workforce is a critical determinant of success. By investing in comprehensive, inclusive, and continuous education and actively involving nurses in the development process, health care leaders can harness the full potential of AI. This will ensure that these powerful technologies act as supportive tools that augment the clinical judgment and compassionate care provided by nurses, ultimately leading to enhanced patient outcomes and the realization of a technologically advanced, efficient, and resilient health care system as envisioned in the Saudi Vision 2030.

## Abbreviations

AI: artificial intelligence
CCN: critical care nurse
GAAIS: General Attitudes Toward Artificial Intelligence Scale
TAM: technology acceptance model

## References

[1] Alnomasy N, Pangket P, Alreshidi B (2025). Artificial intelligence in health care: assessing impact on professional roles and preparedness among hospital nurse leaders. Digit Health.

[2] Bajwa J, Munir U, Nori A, Williams B (2021). Artificial intelligence in healthcare: transforming the practice of medicine. Future Healthc J.

[3] Collins C, Dennehy D, Conboy K, Mikalef P (2021). Artificial intelligence in information systems research: a systematic literature review and research agenda. Int J Inf Manage.

[4] Hendy A, Ibrahim RK, Abdelaliem SM (2025). Supervised machine learning for classification and prediction of stunting among under-five Egyptian children. BMC Pediatr.

[5] Jiang F, Jiang Y, Zhi H (2017). Artificial intelligence in healthcare: past, present and future. Stroke Vasc Neurol.

[6] Topol EJ (2019). High-performance medicine: the convergence of human and artificial intelligence. Nat Med.

[7] Saudi Vision 2030.

[8] AlWatban N, Othman F, Almosnid N, AlKadi K, Alajaji M, Aldeghaither D, Kozlakidis Z, Muradyan A, Sargsyan K (2024). Digitalization of Medicine in Low- and Middle-Income Countries: Paradigm Changes in Healthcare and Biomedical Research.

[9] Alrashedi H, Alnomasy N, Saleh KA, Lamine H, Alkubati SA (2025). The mediating effect of moral distress on the relationship between work environment and depression among critical care nurses. BMC Nurs.

[10] Shimabukuro DW, Barton CW, Feldman MD, Mataraso SJ, Das R (2017). Effect of a machine learning-based severe sepsis prediction algorithm on patient survival and hospital length of stay: a randomised clinical trial. BMJ Open Respir Res.

[11] Robert N (2019). How artificial intelligence is changing nursing. Nurs Manage.

[12] Barchielli C, Marullo C, Bonciani M, Vainieri M (2021). Nurses and the acceptance of innovations in technology-intensive contexts: the need for tailored management strategies. BMC Health Serv Res.

[13] Ronquillo CE, Peltonen LM, Pruinelli L (2021). Artificial intelligence in nursing: priorities and opportunities from an international invitational think-tank of the nursing and artificial intelligence leadership collaborative. J Adv Nurs.

[14] Atalla AD, El-Gawad Mousa MA, Hashish EA, Elseesy NA, Abd El Kader Mohamed AI, Sobhi Mohamed SM (2025). Embracing artificial intelligence in nursing: exploring the relationship between artificial intelligence-related attitudes, creative self-efficacy, and clinical reasoning competency among nurses. BMC Nurs.

[15] Buchanan C, Howitt ML, Wilson R, Booth RG, Risling T, Bamford M (2020). Predicted influences of artificial intelligence on the domains of nursing: scoping review. JMIR Nurs.

[16] Ibrahim AM, Zoromba MA, Abousoliman AD (2025). Ethical implications of artificial intelligence integration in nursing practice in Arab countries: literature review. BMC Nurs.

[17] Alnawafleh KA, Almagharbeh WT, Alfanash HA (2025). Exploring the ethical dimensions of AI integration in nursing practice: a systematic review. J Nurs Regul.

[18] O’Connor S, Yan Y, Thilo FJ, Felzmann H, Dowding D, Lee JJ (2023). Artificial intelligence in nursing and midwifery: a systematic review. J Clin Nurs.

[19] Ramadan OME, Alruwaili MM, Alruwaili AN, Elsehrawy MG, Alanazi S (2024). Facilitators and barriers to AI adoption in nursing practice: a qualitative study of registered nurses’ perspectives. BMC Nurs.

[20] Seibert K, Domhoff D, Bruch D (2021). Application scenarios for artificial intelligence in nursing care: rapid review. J Med Internet Res.

[21] Swed S, Alibrahim H, Elkalagi NK (2022). Knowledge, attitude, and practice of artificial intelligence among doctors and medical students in Syria: a cross-sectional online survey. Front Artif Intell.

[22] Schepman A, Rodway P (2023). The General Attitudes towards Artificial Intelligence Scale (GAAIS): confirmatory validation and associations with personality, corporate distrust, and general trust. Int J Hum Comput Interact.

[23] Davis FD (1989). Perceived usefulness, perceived ease of use, and user acceptance of information technology. MIS Q.

[24] El-Ashry AM, Al Saleh NS, AlOtaibi NG (2025). The impact of artificial intelligence attitudes and acceptance on critical thinking motivation among nursing students in Saudi Arabia. SAGE Open Nurs.

[25] El-Gazar HE, Zoromba MA, Fayed SM (2024). Nurturing success: e-learning readiness and academic self-efficacy in nursing students. BMC Nurs.

[26] Prensky M (2001). Digital natives, digital immigrants part 1. On Horizon.

[27] Cecconi C, Adams R, Cardone A (2025). Generational differences in healthcare: the role of technology in the path forward. Front Public Health.

[28] Shim J, Park DI (2023). The influence of gender equity in nursing education programs on nurse job satisfaction. Healthcare (Basel).

[29] Zhang X, Yang L, Bai Y, Zhang L (2025). Postgraduate nursing students’ knowledge, attitudes, and practices regarding artificial intelligence: a qualitative study. BMC Med Educ.

[30] Salameh B, Eddy LL, Batran A, Hijaz A, Jaser S (2019). Nurses’ attitudes toward the use of an electronic health information system in a developing country. SAGE Open Nurs.

[31] Ibrahim S, Donelle L, Regan S, Sidani S (2019). Predicting registered nurses’ behavioural intention to use electronic documentation system in home care: application of an adapted unified theory of acceptance and use of technology model. Nurs Leadersh (Tor Ont).

[32] Dornan M (2025). Every nurse an AI nurse: a framework for integrating artificial intelligence across nursing practice, education, research and policy. Digit Health.

